# Revealing plasma membrane protein dynamics in living plant cells with single-molecule tracking

**DOI:** 10.1093/jxb/eraf417

**Published:** 2025-09-23

**Authors:** Sven zur Oven-Krockhaus, Leander Rohr, Luiselotte Rausch, Klaus Harter

**Affiliations:** Center for Plant Molecular Biology (ZMBP), University of Tübingen, Tübingen 72076, Germany; Institute for Physical and Theoretical Chemistry, University of Tübingen, Tübingen 72076, Germany; Center for Plant Molecular Biology (ZMBP), University of Tübingen, Tübingen 72076, Germany; Center for Plant Molecular Biology (ZMBP), University of Tübingen, Tübingen 72076, Germany; Center for Plant Molecular Biology (ZMBP), University of Tübingen, Tübingen 72076, Germany; Wake Forest University, USA

**Keywords:** Data analysis, nanodomains, nanoscale organization and dynamics, photoconvertible/-activatable fluorophores, plant protein dynamics, plasma membrane, single-particle tracking photoactivated localization microscopy (sptPALM), super-resolution microscopy

## Abstract

The behavior of proteins and other biomolecules in cellular environments is governed by complex molecular processes. Understanding their temporal dynamics and interactions with other biomolecules or cellular structures is essential for elucidating physiological functions. Single-particle tracking photoactivated localization microscopy (sptPALM) has emerged as a powerful single-molecule technique for investigating these processes with exceptional spatial and temporal resolution. In this Expert View, we introduce sptPALM and focus on its application in the plasma membrane of plant cells. Key aspects and advances in the technique, including instrumentation and data analysis, are discussed to equip researchers with the foundational knowledge required to establish and execute sptPALM experiments. Recent studies are highlighted to demonstrate the potential of sptPALM to advance our understanding of molecular dynamics in plant cells.

## Teaser

This review introduces single-particle tracking photoactivated localization microscopy for studying plasma membrane proteins in plants, outlining key applications and practical guidance on instrumentation, probes, and analysis.

## Introduction

Membranes are one of the key structures of cells and involved in many molecular processes (Stryer *et al*., 2019). The plasma membrane (PM) forms a selective barrier between the cell and the extracellular space, a function with particular importance for plants due to their sessile lifestyle. Changes and modifications in the extracellular environment can be sensed via PM components that perceive, transduce, and translate external signals into adequate cell responses (Gronnier *et al*., 2018).

Today’s notion of a heterogenous membrane architecture originated primarily from the fluid mosaic model (Singer and Nicolson, 1972) that was later expanded by concepts like ‘lipid rafts’—postulated to serve as platforms for interactions between signaling proteins and effector molecules (Simons and Ikonen, 1997).

Many PM-regulated processes happen on a molecular scale, which makes the understanding of events like the interactions of single biomolecules a prime target for research. In this context, the term ‘nanodomain’ was introduced to describe the dynamic, heterogeneous PM organization into distinct domains. To address the long-standing lack of a common definition, a nomenclature guideline was published recently, defining a nanodomain as a distinct PM environment with local accumulation of specific biomolecules, composed of proteins and lipids (Jaillais *et al*., 2024). Moreover, the authors differentiate between the terms ‘nanodomain-organized’ (for molecular clusters) and ‘nanodomain-localized’ (for molecules that associate with pre-existing structures). We adopted their terminology throughout this Expert View.

Multiple studies suggest the existence of pre-formed nanodomain-organized clusters in the PM, essential for signaling events (Bücherl *et al*., 2017; Glöckner *et al*., 2022; Wang *et al*., 2022). Although some clusters share components, it is evident that distinct complexes are spatially separated for specific functions (Bücherl *et al*., 2017; Glöckner *et al*., 2022). While some components, interactions, and functions of such clusters are relatively well studied (Koller and Bent, 2014; Glöckner *et al*., 2022; Wang *et al*., 2022), little attention has been given to the complex functional implications of multimeric protein association within a given cluster. To decipher the properties of nanodomains and their constituent molecules at the nanoscale, advanced methods are needed.

Super-resolution microscopy (SRM), a powerful form of fluorescence microscopy, providing the required spatiotemporal resolution to analyze molecular activities with single-molecule sensitivity (Bond *et al*., 2022). The tracking of single molecules is particularly effective to elucidate dynamic processes at nanoscale resolution (Manzo and Garcia-Parajo, 2015; Wang *et al*., 2021; Nguyen *et al*., 2023; Simon *et al*., 2024), with transformative advances primarily achieved in mammalian-based research. Although plant cells pose certain imaging challenges (Moreno *et al*., 2006; Iwatate *et al*., 2020), methodical advances have helped to overcome initial obstacles, making single-molecule tracking a valuable tool in plant cell research (Cui *et al*., 2018; Bayle *et al*., 2021). Their implementation ranges from the first successful application in plants (Hosy *et al*., 2015) to studies on the influence of nanodomain partitioning on signaling events (Gronnier *et al*., 2017), and uncovering ligand-triggered nanodomain formation as a mechanism for active signaling (Smokvarska *et al*., 2023).

In this Expert View, we familiarize the reader with the concepts and components for setting up dynamic single-molecule experiments, with focus on plant PM proteins. We will discuss recent developments in instrumentation and analysis, as well as provide examples from plant research (Box 1) to underscore the relevance and unique opportunities that this cutting-edge method can offer.

Box 1. **Key developments in sptPALM procedures in plants.**

- Hosy *et al*. (2015) conducted the first successful sptPALM measurements in plant cells. They fused three proteins (*At*PIP2;1, LTi6a, and *At*TIP1;1) to the photoconvertible protein mEos2 and analyzed their diffusion coefficients in the PM of *A. thaliana* root cells, uncovering distinct mobility patterns. Additionally, drug treatments confirmed the feasibility of using sptPALM to study protein dynamics in plant tissues. Beyond demonstrating proof-of-concept, the authors established the foundational sptPALM framework for characterizing protein mobility in plants.
- Gronnier *et al*. (2017) applied sptPALM to examine the nanoscale organization and function of the PM-localized protein *St*REM1.3 in plasmodesmata regulation during pathogen attack. Using Voronoi tessellation, they analyzed nanodomain properties, marking the first use of this cluster analysis algorithm for PM-resident proteins in plants. Their findings revealed differences in cluster size and PM area coverage between wild-type and mutant forms, providing new insights into how protein mobility and nanoscale organization can influence functional outcomes.
- Bayle *et al*. (2021) addressed the lack of unified protocols and method-specific guidance for sptPALM in plants. They provided a comprehensive step-by-step protocol covering experimental preparation, microscopy configuration, image acquisition and data analysis. Emphasizing the importance of proper controls, they detailed thresholds and refinements for data evaluation and discussed the advantages and limitations of sptPALM. This work filled a critical gap, introducing standardized practices and making the method more accessible to the plant science community.
- Smokvarska *et al*. (2023) demonstrated how protein clustering and mobility at the PM influence ROS signaling, focusing on the role of the cell wall sensor FER in regulating PS distribution. The localization of PS by FER was shown to be essential for ROP6 retention in nanodomains and its function in ROS signaling under osmotic stress, which uncovered a new function of FER. This work marks a milestone, emphasizing the value of sptPALM in linking nanoscale protein dynamics to plant cellular function.
- Rohr *et al*. (2024a, Preprint) introduced a dual-color approach to sptPALM in plants using the photoactivatable fluorophores PA-GFP and PATagRFP. Control experiments with identical PM-localized proteins showed comparable mobility parameters, confirming that the different fluorophores did not interfere with the results. Overcoming the long-standing single-channel limitation of sptPALM plant experiments, this technique allows the simultaneous observation of two proteins within the same plant cell and opens new possibilities for studying complex processes such as signal transduction and protein complex formation at the PM.

**Figure:**
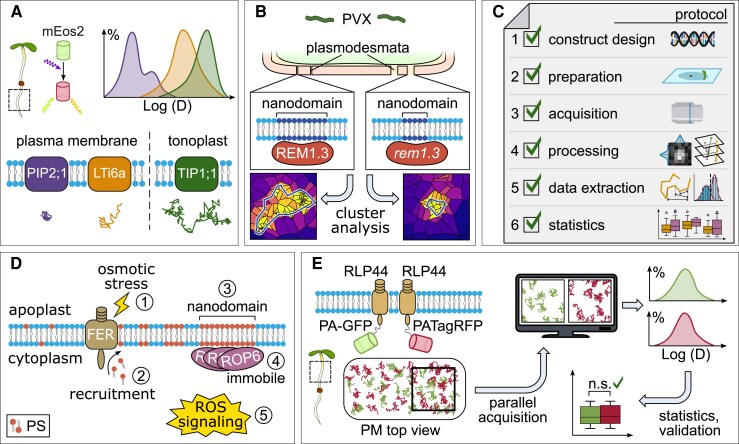

## Visualization of single-molecule dynamics at the plasma membrane

Classical light microscopy had long been confined by the diffraction limit, restricting resolution to 200–250 nm. This changed with the advent of SRM that achieves spatial resolution down to 20–50 nm (Klar *et al*., 2000; Betzig *et al*., 2006; Hess *et al*., 2006; Rust *et al*., 2006). With its versatile molecular labeling techniques, SRM enables precise targeting of individual biomolecules, providing greater specificity while better preserving native structures. The key advantage of SRM is its applicability to live-cell imaging, enabling real-time observation of dynamic biological processes at the nanoscale.

The PM is a primary target for the study of such processes, aiming to observe how individual biomolecules move and interact, and to capture how molecular processes unfold into functional changes at the PM. Due to the high density of biomolecules in the PM, traditional fluorescent labeling usually does not allow to distinguish single molecules. However, single-particle tracking photoactivated localization microscopy (sptPALM) addresses this issue by using special, switchable fluorophores to control their visible density, which enables the tracking of individual tagged PM molecules with high precision (Manley *et al.*, 2008, 2010). Total internal reflection fluorescence microscopy (TIRFM), a specialized widefield microscopy technique, is used to limit the laser illumination into the sample to about 150 nm (Axelrod, 1981, 2001), which can differentiate the PM from other cell compartments, providing the high contrast necessary to detect single fluorophores. In plant cells, however, the additional cell wall layer and its refractive interface with the cytosol interferes with the application of traditional TIRFM (Langhans and Meckel, 2014). To address this, the alternative excitation methods variable-angle epifluorescence microscopy (VAEM) and highly inclined and laminated optical sheet (HILO) have been developed (Konopka and Bednarek, 2008; Tokunaga *et al*., 2008; Seckler *et al*., 2023). These methods reduce background signals from deeper cell layers while still allowing selective PM imaging, albeit at the cost of some optical contrast. While this practical technological adaptation facilitates single-molecule imaging in the complex optical environment of plant tissues, sample thickness remains a limiting factor, effectively restricting sptPALM to epidermal cells in accessible organs such as cotyledons and young leaves, hypocotyls, and roots. Importantly, PM imaging is limited to the region closest and parallel to the coverslip, excluding lateral and basal domains that may be critical for studying membrane polarity – though extensive and valuable insights can be gained from the accessible apical surface in many contexts.

Combined with selective illumination techniques, sptPALM is a true single-molecule technique that allows to quantitatively ‘resolve the dynamics of individual molecules by tracking them in live cells’ (Manley *et al*., 2008). In contrast, TIRFM methods that are based on traditional fluorophores like EGFP typically capture the movement of domains or molecule clusters (Martinière and Zelazny, 2021).

## Instrumentation

While several commercial systems support SRM, access may still be limited by high costs or restricted configurability. Moreover, both commercial and custom setups often require specialized personnel for operation and data analysis which can present an additional barrier (Munck *et al*., 2024). Fortunately, accessibility has improved in recent years, driven by ‘the proliferation of versatile open software and hardware for microscopy’ (Eisenstein, 2021), alongside more affordable lasers and detectors. As a result, many researchers now implement custom setups tailored to their experimental needs.

A minimal configuration for sptPALM includes a high-numerical-aperture (≥1.45 NA) objective, laser-based excitation, and a sensitive camera with high frame rates and low noise. Both EMCCD and modern sCMOS cameras are widely used—while older sCMOS sensors may require calibration for pixel-level noise, newer models generally offer high performance without correction (Power *et al*., 2024). Plant-specific challenges, such as tissue thickness and autofluorescence, require additional considerations, including suitable illumination strategies (see last section), appropriate emission filters, and environmental control for live-cell imaging.

Researchers without prior experience may benefit from collaborating with experienced groups in optical system design. Several recent publications offer detailed, open-source guidance for implementing sptPALM, including hardware design and software control (Danial *et al*., 2022; Deschamps *et al*., 2023; Niederauer *et al*., 2023; Power *et al*., 2024).

## Fluorophores for sptPALM

Tracking single molecules in plant cells is more challenging than in animal systems, in part because the plant cell wall can hinder the reliable uptake of organic dyes commonly used in animal cells (Magrassi *et al*., 2019; Iwatate *et al*., 2020; Lelek *et al*., 2021). As a result, genetically encoded fluorescent proteins (FPs) remain the most widely used labeling strategy for sptPALM in plants. While natural blinking behavior of standard FPs, such as mCitrine, has been explored as an alternative for single-molecule analysis (Fuchs *et al*., 2021, Preprint), most sptPALM applications rely on fluorophores whose fluorescence can be precisely regulated by specific wavelengths of light to achieve the sparse emitter density required. This includes photoswitchable, photoconvertible, and photoactivatable FPs (Box 2), of which only the latter two are widely used in sptPALM studies. For photoconvertible FPs, plant researchers have exclusively relied on the exceptionally bright and stable mEos variants, starting with the pioneering work of Hosy *et al*. (2015). As the photoconversion mode of mEos spans two major spectral areas, this limits its application to single-protein analyses. However, the simultaneous analysis of multiple proteins is essential for deciphering the intricate physiological processes of plants (McWhite *et al*., 2020), necessitating the use of additional spectrally separable FPs like the photoactivatable green fluorescent protein (PA-GFP) and photoactivatable Tag red fluorescent protein (PATagRFP) (Box 2). Although less commonly used, PA-GFP is applied to plant research (Martinière *et al*., 2012; McKenna *et al*., 2019; Rohr *et al*., 2024b), and its simultaneous use with PATagRFP in plant PM analysis has been demonstrated recently in a dual-color approach (Rohr *et al*., 2024a, Preprint).

Box 2. **Photoactivatable Fluorescent Proteins for sptPALM in plant research.**

- The principle of photoactivation involves the transition of a fluorescent protein (FP) from a non-fluorescent (OFF) state to a fluorescent (ON) state when exposed to specific wavelengths of light. In the case of PATagRFP (Subach *et al*., 2010), the OFF state is largely non-excitable by the imaging laser, ensuring minimal background fluorescence in the imaging channel. Activation by UV light triggers a multistep photo-oxidation reaction that extends the π-electron system of the chromophore, allowing it to absorb at the excitation wavelength of the imaging laser and emit fluorescence. In ensemble measurements, the fraction of activated molecules can be adjusted by tuning the activating laser intensity, ensuring sufficient spatial separation of emitters in sptPALM.
- Irreversible photoactivatable FPs, such as PA-GFP (Patterson and Lippincott-Schwartz, 2002) and PATagRFP (Subach *et al*., 2010), exist natively in a non-fluorescent state and are activated by UV irradiation. Once activated, these FPs exhibit distinct fluorescence emission profiles when excited by the appropriate imaging lasers, making them suitable for use in the appropriate color channels. Their non-fluorescent off-states and spectrally distinct emission wavelengths allow their combination in dual-color experiments without spectral overlap. In contrast, the irreversibly photoconvertible FP mEos3.2 (Zhang *et al*., 2012) exists natively in a green-fluorescent form that can be converted to a red-fluorescent form upon UV irradiation. After conversion, excitation with the imaging laser selectively excites the red form, ensuring that only photoconverted molecules are detected in the red channel. However, due to the native fluorescent green form, the non-converted population of mEos FPs preclude its simultaneous use with the green-emitting PA-GFP in dual-color experiments. Otherwise, the mEos variants mEos2 (McKinney *et al*., 2009) and mEos3.2 (Zhang *et al*., 2012) are preferred for sptPALM applications in plant research due to their robust performance in single-molecule imaging. The table below summarizes key characteristics of PA-GFP, PATagRFP (activated forms), and mEos3.2 (converted form), as reported in their original publications. The molar extinction coefficient (ε) corresponds to the excitation maximum; brightness is calculated as the product of ε and quantum yield. These parameters provide a useful basis for comparing fluorophore performance in sptPALM applications.

**Figure:**
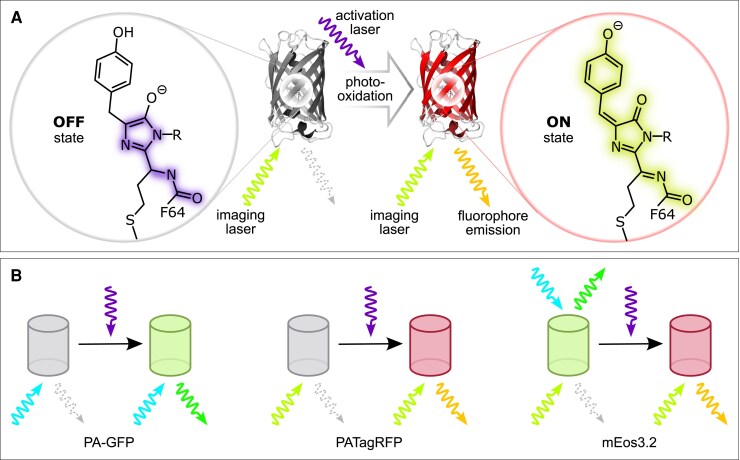

**Table:** 

Fluorophore	Excitation maximum (nm)	Emission maximum (nm)	Quantum yield	ɛ (M^−1^cm^−1^)	Brightness (mM^−1^cm^−1^)	pH stability (pK_a_)	Photostability t_½_ (s)
PA-GFP	504	517	0.79	17 400	14	n.d.	n.d.
PATagRFP	562	595	0.38	66 000	25	5.3	180
mEos3.2	572	580	0.55	32 200	18	5.8	48.0

In addition, recent developments in probe technologies are emerging as valuable complements to fluorescent proteins for plant imaging. Optimized HaloTag systems have enabled singleparticle tracking in live plant cells with improved labeling performance (Qian *et al*., 2025). Separately, a new family of ‘bridged’ dyes have shown greatly enhanced photostability compared to existing dyes in plant tissues and are expected to support advanced applications such as multiplexed single-molecule tracking (Zhang *et al*., 2025).

Autofluorescence from plant tissues demands careful selection of bright, stable fluorophores with good signal-to-noise performance. The FPs discussed here are well validated for plant systems and, when paired with modern localization algorithms (see next section), typically allow reliable single-molecule detection.

## Analysis of sptPALM data

### Localization

**Fig. 1.**
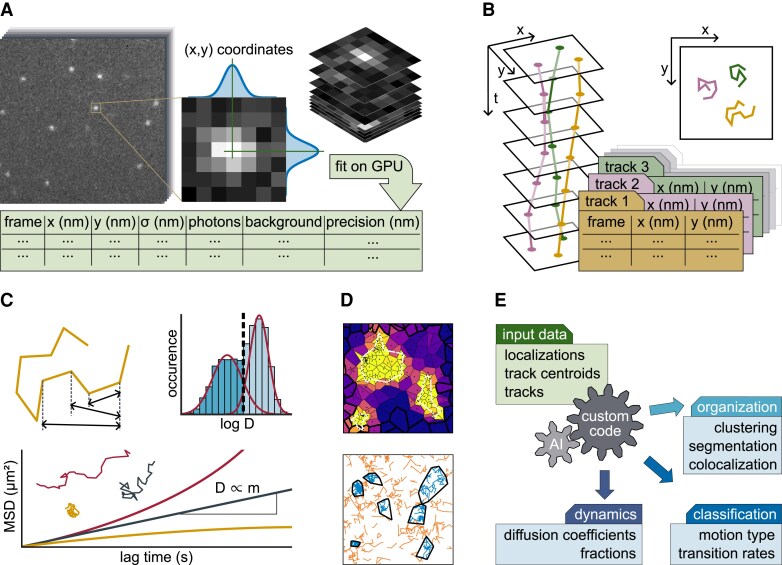
Workflow of single-particle tracking photoactivated localization microscopy (sptPALM) analysis. (A) Localization: single molecule spots are cut out of the raw data image stack, fitted with two-dimensional Gaussian functions and the results (most importantly, their coordinates) stored as lists. (B) Tracking: single-molecule positions are grouped to tracks, based on their relative positions in subsequent frames. (C) Mean square displacement (MSD) analysis: the average squared distance the molecules have traveled in certain time intervals (top left) can be plotted in MSD curves (bottom), showing a straight line for free Brownian motion (dark gray), for which the slope is directly proportional to the diffusion coefficient. Other motion types like superdiffusive (red) or subdiffusive (yellow) show different curve shapes (inset: corresponding single-molecule example tracks). Individual MSD curve fitting for single molecules allows to plot diffusion coefficient distributions (top right histogram), revealing populations with different motility that are obscured in ensemble measurements. (D) Cluster analysis: the extraction of structural parameters from sptPALM data can be based on localizations, track centroids (e.g. top, Voronoi tessellation) or entire tracks (e.g. bottom, nanoscale spatiotemporal indexing clustering (NASTIC)). (E) Overview of analysis modules for single molecule data. Community-driven software and algorithms offer diverse and complex analysis methods, which are increasingly incorporating artificial intelligence (AI) features.

The stochastic activation of fluorophores in sptPALM ensures that the density of visible fluorophores is sufficiently sparse to isolate single emitters (Lelek *et al*., 2021). Knowledge of the single fluorophore emission pattern enables the calculation of their original position. For optimal localization accuracy (Thompson *et al*., 2002), the optical system is usually adjusted to cover 100-160 nm in the sample plane per camera pixel, projecting each fluorophore to 2-3 pixels and allowing emitter centers to be localized within 10–20 nm. For simple 2D cases, the localization fit function is usually a 2D Gaussian, allowing to extract additional parameters such as the size of the emitter point spread function, the number of emitted photons and the localization precision (Fig. 1A).

### Tracking

Following localization, the next step is to track the emitters over time (Fig. 1B) by linking molecule positions that remain within a defined distance in successive images. Advanced algorithms use global optimizers and can also cover track branching or merging. Due to the typically low fluorophore density in sptPALM, a fast and straightforward tracking algorithm (Jaqaman *et al*., 2008) is often used that also bridges short gaps where molecules are temporarily undetectable (Chenouard *et al*., 2014).

### Extraction of single-molecule dynamics

The mean square displacement (MSD) function remains the most common basis (Spagnolo and Luin, 2024) to extract diffusion coefficients and movement patterns from sptPALM data. The average MSD is computed across all tracks in a sample and represents the average squared distance the molecules have traveled over increasing time intervals. The diffusion coefficient is typically estimated by applying a linear fit to the initial points of the MSD curve. Single-molecule data also allows for a more detailed analysis by estimating diffusion coefficients for each individual track. Plotting the distribution of these values as a histogram can reveal non-uniform mobility patterns that would be obscured in the overall MSD average (Fig. 1C). Other algorithms bypass classical track building and obtain quantitative dynamic parameters via the temporal analysis of relative distances between all localizations in the sample (Martens *et al*., 2024).

The dynamics of biomolecules in dense environments such as the PM are more accurately described by so-called anomalous diffusion. This behavior involves transitions between different motion states or patterns, which can challenge the traditional use of MSD analysis (Simon *et al*., 2024). Advanced analytical methods can decompose tracks into segments based on their diffusion type and then allow for evaluation of spatiotemporal heterogeneities in the environment of individual molecules. Analyses that consider anomalous diffusion can provide much deeper insights into the dynamics of individual proteins under physiological conditions, often taking advantage of artificial intelligence (AI) (see Box 3). In general, longer single-molecule tracks make such analyses more reliable. A recent study in plants presented a method of extending track durations that, when combined with machine learning, enabled the detection of transient arrest events (von Arx *et al*., 2024, Preprint).

Box 3. **Advanced analyses of dynamics in single-molecule tracking experiments.**

Diffusing biomolecules in living cells often deviate from standard Brownian motion, due to their numerous and complex interactions with cellular structures and environments. The analysis of this so-called anomalous diffusion therefore allows the corresponding biomolecular interactions to be analyzed in greater depth (Simon *et al*., 2024).

For example, the analysis of jump distance distributions can be used to draw conclusions as to whether other movement patterns such as confinement or directed motion are present in addition to free diffusion (Schütz *et al*., 1997). Furthermore, individual molecular tracks can also be analyzed for the change between different motion patterns, e.g. via Bayesian inference (Krog *et al*., 2018), Hidden Markov models (Das *et al*., 2009), moment scaling spectra (Ferrari *et al*., 2001), or diffusion distribution analysis (Vink *et al*., 2020). The decomposition of tracks into segments based on their diffusion type then also allows to evaluate spatiotemporal heterogeneities in the environment of individual molecules.

Particular attention should be paid to the rapid integration of artificial intelligence (AI) into all these processes. Many algorithms benefit significantly from machine learning and deep learning, and their development and accessibility has been promoted by numerous publications in recent years. A key challenge in SMLM experiments is the detection of single-molecule signals amidst variable background noise in biological samples. With the help of AI, even noisy data can be handled efficiently, and localization precision can be improved (Speiser *et al*., 2021). In tracking, AI can reduce errors in assembling tracks from dense localization data (Spilger *et al*., 2021). Because AI excels at pattern recognition, it can be applied to classify diffusive motion types and identify transitions between them, which is especially beneficial to analyze anomalous diffusion (Kowalek *et al*., 2019; Chen *et al*., 2021).

### Extraction of structural domain parameters

While it is accepted that molecular assemblies in the PM are essential for the initiation of signaling, their precise functional classification often remains challenging to determine (Jaillais *et al*., 2024). Therefore, assembly parameters such as size, number, temporal progression, and functional relationships are key targets to unravel their role in the PM. Single-molecule data from techniques such as sptPALM provide valuable insights, especially through so-called cluster analysis methods (Fig. 1D). These include tools like ClusterViSu (Andronov *et al*., 2016), SR-Tesseler, and its updated platform PoCA (Levet *et al*., 2015; Levet and Sibarita, 2023). These methods rely on a high number of localizations to perform well but are limited as sptPALM requires low localization numbers for unambiguous track-building. However, track data can also be directly used for cluster analysis using spatial indexing (Wallis *et al*., 2023) or to generate spatial maps of heterogeneous motion parameters with Bayesian inference (El Beheiry *et al*., 2015). Cluster analysis of sptPALM data is still an emerging field, where the community has not yet agreed on a standard method or even objective choices of input parameters for the various algorithms used. However, when using a specific clustering algorithm with constant input parameters, qualitative differences such as growing or shrinking cluster sizes can be revealed in comparative experiments. As with the analysis of single-molecule dynamics, artificial intelligence (AI)-based cluster analysis methods will play a crucial role in the future (Hyun and Kim, 2023).

### Software for sptPALM data analysis

Many research groups develop their own sptPALM analysis pipelines, and numerous algorithms and free software solutions can be found in literature (Fig. 1E). For single-molecule localization, various plugins have been developed for ImageJ/Fiji (van de Linde, 2019) and new software solutions are constantly being published (Sage *et al*., 2019). Also, comprehensive programs continue to emerge for particle tracking (Simon *et al*., 2024) that calculate dynamic parameters from the localization data (Pineda *et al*., 2023; Wolf *et al*., 2023; Martens *et al*., 2024) or single-molecule trajectories (Slator *et al*., 2015; Relich *et al*., 2016; Hansen *et al*., 2018; Lindén and Elf, 2018; Vink *et al*., 2020; Chen *et al*., 2021; Karslake *et al*., 2021; Simon *et al*., 2023).

However, few software packages provide comprehensive guidance through all analytical processes or cut through the vast array of available methods. Thus, first-time users face challenges to use or create appropriate analysis software without advanced coding expertise. At the same time, a streamlined, freely available tool that combines the most relevant algorithms is considered critical for non-experts to use SRM (Hugelier *et al*., 2023). We recently developed a software package that meets these requirements, integrating key steps like localization, tracking, MSD analysis, and cluster analysis into structured processes, and is easily accessible and workable even for SRM novices (Rohr *et al*., 2024b). More complex approaches, such as anomalous diffusion methods, have recently been evaluated (Muñoz-Gil *et al*., 2021, 2025) and necessitate specialized software solutions (also see Box 3). Most of these can import localization and track data provided by the more general software mentioned above.

### Investigations with sptPALM in the plant PM

The first successful application of sptPALM in plant cells (Hosy *et al*., 2015) was a major breakthrough for this technique in the plant field (Box 1A). In a collaboration of plant researchers and neuroscientists with a broad interest in SRM, three mEos2 fusion proteins were expressed in *A. thaliana* root epidermis cells: *At*PIP2;1 (aquaporin), LTi6a (PM-intrinsic protein) and *At*TIP1;1 (tonoplast-intrinsic protein). By tracking individual molecules, the authors revealed distinct mobility properties across the different protein fusions. They found that *At*PIP2;1 was nearly immobile, which might be a plant-specific property when compared with mammalian aquaporin analogues. In contrast, LTi6a and *At*TIP1;1 exhibited much faster mobility, with the latter representing ’the fastest motion ever recorded for a membrane protein‘ according to the authors. The high resolution of sptPALM also revealed distinct mobility fractions of *At*PIP2;1. Notably, the more mobile fraction increased in response to both actin depolymerization and osmotic exposure-induced plasmolysis, suggesting that actin and the cell wall are involved in the confinement of some plant membrane proteins.

Two years later, Gronnier *et al*. (2017) applied plant sptPALM studies in a more functional context (Box 1B), characterizing a Group 1b REMORIN from *Solanum tuberosum* (*St*REM1.3), which had previously shown to localize to the PM and form nanodomain-organized structures (Demir *et al*., 2013; Raffaele *et al*., 2013). The C-terminal anchor of REM (REM-CA) is critical for PM-targeting and required for the *St*REM1.3-caused restriction of virus movement between plant cells by decreasing plasmodesmata permeability (Raffaele *et al*., 2009; Perraki *et al*., 2012; Raffaele *et al*., 2013; Konrad *et al*., 2014; Perraki *et al*., 2014; Gui *et al*., 2015). In their sptPALM experiments, both the wild type and *St*REM1.3 mutants were expressed in *N. benthamiana* leaf cells, exhibiting confined diffusion, with the mutants showing lower mobility. Cluster analysis based on the sptPALM data revealed distinct nanodomain size differences between mutants. In comparison with the wild type, the relative PM area occupied by mutant clusters was reduced, but no clear trends were observed for the average number of proteins in clusters or their densities. These findings suggest that protein mobility can be uncoupled from supramolecular organization, as shown by variants with lower mobility but larger clusters.

In 2021, Bayle and colleagues addressed the lack of a comprehensive practical guide for sptPALM experiments in plants. In their detailed publication (Bayle *et al*., 2021), the authors discussed the advantages and limitations of sptPALM compared to other techniques for analyzing PM protein diffusion properties and presented a step-by-step protocol that also includes microscope hardware considerations (Box 1C). By offering recommended acquisition parameters, troubleshooting tips, proper control strategies, and guidance on data analysis, the work of Bayle and colleagues introduced the sptPALM approach to the broader plant science community.

Single-molecule tracking has since been applied to various biological questions in plant systems. Smokvarska *et al*. (2023) explored the role of FERONIA (FER) in Rho-of-plants (ROP) signaling under hyperosmotic stress in *A. thaliana*. Previous studies had shown that ROP6 localizes to PM nanodomains (Smokvarska *et al*., 2020), which accumulate effector proteins for reactive oxygen species (ROS) production. Considering that phosphatidylserine (PS) regulates ROP6 retention (Platre *et al*., 2019), Smokvarska and colleagues found that FER affects the quanitity and/or availability of PS at the PM, potentially modulating ROP signaling (Box 1D). To uncover the underlying molecular processes, they used sptPALM, as ROP6 dynamics and nanodomains cannot be resolved by conventional microscopy. Previous experiments showed that ROP6 exists as both a mobile and immobile fraction at the PM (Smokvarska *et al*., 2020), with the immobile fraction increasing under osmotic stress. In a *fer* mutant background, ROP6 nanodomains still form but the increase in the immobile fraction upon osmotic treatment is no longer observed. Additional experiments with the FER ligand RAPID ALKALINIZATION FACTOR 23 (RALF23) revealed application-induced changes in ROP6 nanodomain size and density, likely tied to an altered PS distribution. Their findings propose a model in which FER regulates PS distribution to fine-tune ROP6 dynamics and downstream signaling. Beyond these insights, Smokvarska and colleagues highlighted the transformative potential of sptPALM, establishing it as a vital tool for connecting nanoscale protein organization and dynamics to cellular function.

Rohr *et al*. (2024a, Preprint) advanced sptPALM for plants by introducing a dual-color approach (Box 1E). While PA-GFP had already proven effective in plant cells (McKenna *et al*., 2019), pairing it with a codon-optimized version of PATagRFP (Subach *et al*., 2010) provided a spectrally complementary system. Both photoactivatable FPs, fused to proteins of interest, were tested individually in transient *N. benthamiana* transformations and transgenic *A. thaliana* seedlings, performing well in both systems (Rohr *et al*., 2024b). Subsequent dual-color experiments in transgenic *A. thaliana* allowed the simultaneous observation of the same protein fused to either PA-GFP or PATagRFP within the same cell, with both fusions exhibiting comparable diffusion coefficients and a similarly heterogeneous spatial PM distribution (Rohr *et al*., 2024a, Preprint). These results align with previous single-channel studies using mEos variants (Jaillais and Ott, 2020; Rohr *et al*., 2024b). The dual-color approach provides the ability to directly compare the behavior of two different proteins in the same cellular context. Consequently, it minimizes variability and enables more robust studies of protein dynamics and interactions at the nanoscale, offering significant new opportunities for the plant science community to explore complex cellular processes.

## Conclusion and perspectives

The application of sptPALM in plant research is still emerging, but examples from both early studies and recent advances underscore its significant potential to enhance our understanding of molecular dynamics and spatial organization at the subcellular level. While we have focused on the plasma membrane, the utility of sptPALM extends to other cellular compartments and organelles, as demonstrated by its application to the endoplasmic reticulum (Pain *et al*., 2024) and tonoplast (Hosy *et al*., 2015). Additional targets such as cytoskeletal or cell wall-associated proteins have also been suggested as promising candidates for future applications (Bayle *et al*., 2021).

Several key challenges remain. Multicolor imaging is essential for studying biomolecular interactions in plants, but its adoption is still constrained by the limited availability of spectrally compatible and photostable probes. Recent advances, such as optimized HaloTag systems and next-generation dyes, offer promising multiplexing capabilities that would also benefit from AI-based approaches for spectral unmixing. Additionally, capturing dynamic responses to environmental stimuli will require imaging strategies that minimize photodamage, supported by brighter, more stable fluorophores and sample-preserving technologies like vertical-stage microscopes or microfluidic devices.

Looking forward, sptPALM is poised to contribute to a deeper, systems-level understanding of plant cell biology. Potential avenues include integration with omics-based approaches and experimental strategies that link molecular dynamics to cell physiological outcomes, helping to directly connect single-molecule behavior to functional relevance. Advances in AI-assisted analysis are already making it feasible to extract nuanced, quantitative information on biomolecular dynamics from increasingly large and complex datasets. Democratization through modular hardware platforms, improved open-source software, and community-driven protocols will further lower the barriers to adoption.

Ultimately, the broader implementation of sptPALM in plant biology depends on technological accessibility, collaborative expertise, and alignment with key biological questions. Understanding how protein mobility and clustering relate to processes like signal transduction, development, or stress responses will be essential. By integrating quantitative single-molecule data, plant researchers can shape the next generation of studies and gain unprecedented insight into complex signaling events.

## Data Availability

No new data were created or analyzed for this article.
